# Spread of West Nile Virus and Usutu Virus in the German Bird Population, 2019–2020

**DOI:** 10.3390/microorganisms10040807

**Published:** 2022-04-12

**Authors:** Ute Ziegler, Felicitas Bergmann, Dominik Fischer, Kerstin Müller, Cora M. Holicki, Balal Sadeghi, Michael Sieg, Markus Keller, Rebekka Schwehn, Maximilian Reuschel, Luisa Fischer, Oliver Krone, Monika Rinder, Karolin Schütte, Volker Schmidt, Martin Eiden, Christine Fast, Anne Günther, Anja Globig, Franz J. Conraths, Christoph Staubach, Florian Brandes, Michael Lierz, Rüdiger Korbel, Thomas W. Vahlenkamp, Martin H. Groschup

**Affiliations:** 1 Friedrich-Loeffler-Institut, Federal Research Institute for Animal Health, Institute of Novel and Emerging Infectious Diseases, 17493 Greifswald-Insel Riems, Germany; felicitas.bergmann@fli.de (F.B.); cora.holicki@fli.de (C.M.H.); balal.sadeghi@fli.de (B.S.); markus.keller@fli.de (M.K.); martin.eiden@fli.de (M.E.); christine.fast@fli.de (C.F.); martin.groschup@fli.de (M.H.G.); 2 German Center of Infection Research (DZIF), Partner Site Hamburg-Lübeck-Borstel-Riems, 17493 Greifswald-Insel Riems, Germany; 3 Clinic for Birds, Reptiles, Amphibians and Fish, Justus Liebig University Giessen, 35392 Giessen, Germany; dominik.fischer@vetmed.uni-giessen.de (D.F.); michael.lierz@vetmed.uni-giessen.de (M.L.); 4 Department of Veterinary Medicine, Small Animal Clinic, Freie Universität Berlin, 14163 Berlin, Germany; kerstin.mueller@fu-berlin.de; 5 Institute of Virology, Faculty of Veterinary Medicine, Leipzig University, 04103 Leipzig, Germany; michael.sieg@vetmed.uni-leipzig.de (M.S.); vahlenkamp@vetmed.uni-leipzig.de (T.W.V.); 6 Department of Small Mammal, Reptile and Avian Diseases, University of Veterinary Medicine Hannover, 30559 Hannover, Germany; maximilian.reuschel@tiho-hannover.de (M.R.); 7 Seehundstation Nationalpark-Haus Norden-Norddeich, 26506 Norden, Germany; rs@seehundstation-norddeich.de; 8 Wildlife Research Institute, 53229 Bonn, Germany; luisa.fischer@lanuv.nrw.de; 9 Leibniz Institute for Zoo and Wildlife Research (IZW), Department of Wildlife Diseases, 10315 Berlin, Germany; krone@izw-berlin.de; 10 Clinic for Birds, Small Mammals, Reptiles and Ornamental Fish, Centre for Clinical Veterinary Medicine, Ludwig Maximilians University Munich, 85764 Oberschleißheim, Germany; monika.rinder@vogelklinik.vetmed.uni-muenchen.de (M.R.); korbel@lmu.de (R.K.); 11 Wildlife Rescue and Conservation Center, 31553 Sachsenhagen, Germany; karolin.schuette@wildtierstation.de (K.S.); florian.brandes@wildtierstation.de (F.B.); 12 Clinic for Birds and Reptiles, Faculty of Veterinary Medicine, Leipzig University, 04103 Leipzig, Germany; volker.schmidt@vogelklinik.uni-leipzig.de; 13 Friedrich-Loeffler-Institut, Federal Research Institute for Animal Health, Institute of Diagnostic Virology, 17493 Greifswald-Insel Riems, Germany; anne.guenther@fli.de; 14 Friedrich-Loeffler-Institut, Federal Research Institute for Animal Health, Institute of International Animal Health/One Health, 17493 Greifswald-Insel Riems, Germany; anja.globig@fli.de; 15 Friedrich-Loeffler-Institut, Federal Research Institute for Animal Health, Institute of Epidemiology, 17493 Greifswald-Insel Riems, Germany; franz.conraths@fli.de (F.J.C.); christoph.staubach@fli.de (C.S.)

**Keywords:** West Nile virus, Usutu virus, bird, monitoring, flavivirus, Germany

## Abstract

West Nile virus (WNV) and Usutu virus (USUV) are important flaviviruses circulating in Germany. While USUV was first reported more than 10 years ago, WNV has only reached the country in 2018. Wild birds are important amplifying hosts for both viruses. Therefore, we have been monitoring the bird population in different regions of Germany by a previously established network for many years. This report summarizes the results of molecular and/or serological methods of 2345 blood samples from birds of 22 different orders and over 2900 bird carcasses from 2019 and 2020. USUV RNA circulation was found in different regions of Germany, with emphasis on USUV lineages Europe 3 and Africa 3. Increased evidence of USUV lineage Europe 2 was detected in eastern Germany. WNV RNA was found only in birds from the eastern part of the country. The seroprevalence for USUV was between 3.11% and 7.20% in all three regions investigated, whereas the WNV seroprevalence spanned from 14.77% to 16.15% in eastern Germany, with a noticeable tendency for a westward and southward expansion in both years. Thus, wild bird monitoring for WNV and USUV can serve as an early warning system for a human exposure risk.

## 1. Introduction

Wild birds play a key role in the transmission cycle of zoonotic arthropod-borne viruses (arboviruses) [1]. As amplifying hosts, they are often involved in the distribution of arboviruses and, as such, are an ideal target for many monitoring programs. In this regard, special focus is placed on the spread of West Nile virus (WNV) and Usutu virus (USUV) in the bird population throughout many European countries (summarized by [2]). Both arboviruses are primarily transmitted to birds by ornithophilic mosquitoes and to mammals by potential bridge-vectors [3]. Historically, both flaviviruses were confined to the African continent, without association to mortalities in birds and humans. The situation, however, changed with the introduction of both viruses to Europe and the Americas (WNV only) with clinical disease arising in birds and mammals (e.g., in humans and horses for WNV) [4,5]. 

WNV and USUV are positive-sense, single-stranded RNA viruses with spherical and enveloped virions [6]. Both viruses are grouped in the genus Flavivirus, family *Flaviviridae* [7]. Until now, up to nine lineages have been proposed to classify WNV strains [8], of which lineages 1 and 2 represent the most important human pathogens. Isolates of USUV are currently classified into eight lineages (Africa 1–3 and Europe 1–5) [9]. 

In 1996, USUV appeared in Europe for the first time, as confirmed by retrospective studies performed in Italy [10]. Further virus detections were recorded in Austria in 2001 with hundreds of dead Eurasian blackbirds (*Turdus merula*) [11], followed by an outbreak in Hungary in 2005 [12], in Switzerland in 2006 [13], and a striking outbreak in Italy in 2009 [14]. In Germany, USUV was isolated for the first time in 2010 in a pool of mosquitoes (*Culex pipiens pipiens*) collected in Weinheim, south of Frankfurt [15]. The following year was characterized by mass-mortality events in the avifauna (particularly in Eurasian blackbirds) in the north of the Upper Rhine valley and the neighboring regions of the Palatinate and the Neckar valley [16]. Since then, the southwest of Germany has experienced recurring, regionally restricted mortality events particularly in Eurasian blackbirds but also zoo birds, such as owls kept in aviaries [17]. By contrast, only two sporadic cases have been observed in more northern parts of Germany, Bonn and Berlin, with the introduction of new USUV lineages into the country [18,19]. In 2016, an upsurge in the number of USUV cases was recorded in the southwest of Germany, but also in the northwestern and eastern federal states of Saxony, Saxony-Anhalt, and North Rhine-Westphalia [20,21], with virus spreading to neighboring countries to the west [9]. In 2017, a prominent northward drift of USUV was detected in Germany. Since 2018, USUV has been circulating nationwide, covering nearly all federal states, with five USUV lineages present (Africa 2, Africa 3, Europe 2, Europe 3, and Europe 5) [9,22]. At the same time, massive and ongoing USUV epizootics have also been detected in 16 other European countries [23,24,25,26]. Taken together, thousands of Eurasian blackbirds and many captive and free-living owls and other birds have succumbed to USUV infections in Germany since 2011 [27]. 

In Germany, a nationwide bird surveillance network was established years before the first detection of USUV, to systematically monitor zoonotic arbovirus infections (with a focus on USUV and WNV) in migratory and resident birds [28,29]. The network includes veterinary state offices and laboratories of all federal states, veterinary schools (bird clinics, virology departments), zoological gardens and wildlife parks, the Bernhard Nocht Institute for Tropical Medicine (BNITM), the German Working Group for Mosquito Control (KABS), the Nature and Biodiversity Conservation Union (NABU), the Leibniz Institute for Zoo and Wildlife Research (IZW), several private bird clinics and practices, bird sanctuaries, falconry centers, and numerous volunteer ornithologists spread throughout Germany. Therefore, it was possible to follow the spread of USUV over the last 10 years, as well as the invasion of other zoonotic arboviruses into the bird population [17,21,22,30,31,32]. 

WNV has shown an increased occurrence in the Mediterranean region since the mid-1990s, with annually recurring waves of disease in humans, horses, and avifauna [2,4]. The unusually hot weather all over Europe in the summer of 2018, with an extremely long period of high temperatures, may have provided favorable conditions for the incursion and establishment of WNV into new areas and countries [33]. The first WNV outbreak in Germany was identified also in 2018 in the context of this nationwide bird surveillance [34], when WNV was isolated from a great grey owl (*Strix nebulosa*) from the zoological garden in Halle (Saale) in eastern Germany. By the end of 2018, 12 WNV cases in birds and two cases in horses had been confirmed [35]. Phylogeographic analysis revealed that the causative agent was a WNV lineage 2 strain belonging to the central European subclade II, which indicated a single introduction event of WNV into Germany, most likely from the Czech Republic [34]. One year after the first autochthonous transmission of WNV to birds and horses, an epizootic re-emergence of WNV occurred in 2019. The first official bird case was detected at the beginning of July in Berlin, followed by 76 cases in wild and zoo birds until the end of the year [36,37,38]. Moreover, WNV was found for the first time in mosquitoes of the *Culex pipiens* complex, which are known as potential vectors [39]. Furthermore, five human autochthonous cases were diagnosed in regions in the eastern part of the country where WNV had become enzootic [40]. A comprehensive summary and phylogeographic analysis on the WNV epizootic in Germany in 2018 and 2019 demonstrated that Germany experienced several independent WNV lineage 2 introduction events and that strains of a distinct group (Eastern German WNV clade), were predominant in 2018 and 2019 [38]. Extraordinarily high temperatures in 2018 and 2019 allowed a low extrinsic incubation period (EIP), which drove the epizootic emergence and, in the end, most likely triggered the 2019 epizootic [34,38]. The WNV epizootic continued in 2020, when once again numerous birds and horses were affected [41] A total of 65 bird cases were detected in 2020. All birds showed more or less severe clinical signs, and only one golden eagle (*Aquila chrysaetos*) survived the infection. In 2020, the majority of fatal cases in birds was again confined to the known affected regions of eastern Germany (Saxony, Saxony-Anhalt, Brandenburg, and Berlin). However, for the first time, WNV infections were detected in diseased and/or dead zoo and wild birds in the federal state of Thuringia, and a tendency to spread to other districts of Brandenburg was observed [41]. Also in 2020, more than twenty human West Nile fever cases or WNV neuroinvasive disease were recorded, including the first fatal case [42,43]. 

The present study is an in-depth molecular and serological analysis of the spread of USUV and WNV in the German bird population in 2019 and 2020.

## 2. Materials and Methods

### 2.1. Sample Collection

Bird monitoring based on the nationwide wild bird surveillance network for zoonotic arboviruses has been carried out for more than ten years. Through close cooperation with various partners from the avifauna community (described by Michel et al. [22]), it was possible to obtain and examine more than 2300 blood samples from different bird species for the present study (constituting the first monitoring panel or live bird panel). Specifically, these were 977 blood samples in 2019 and 1368 blood samples in 2020 from birds of 20 and 17 different orders, respectively. Birds were categorized as resident birds or captive birds (zoos, sentinels) which remain in their German habitat year-round, partially migratory birds (parts of the population stay in the German habitat and parts migrate), short-distance migratory birds (usually migrating less than 1000 to a maximum of 2000 km and not crossing the Sahara Desert), and long-distance migratory birds (usually migrating more than 3000–4000 km and/or crossing the Sahara Desert). The blood samples were collected throughout the year and were derived from routine hematological or chemical blood analyses of sick and injured birds or were taken immediately following euthanasia in birds with an infaust prognosis. Samples were acquired by puncturing the metatarsal, jugular, or wing vein. By recording individual features (e.g., body weight, markings, morphologic or radiographic characteristics), we tried to largely exclude double sampling. Samples were separated and stored prior to processing: coagulated blood (cruor) at −70 °C and serum at −20 °C.

Our second monitoring panel comprised organ samples from 2976 deceased wild and captive birds; in detail, 1238 carcasses from 2019 and 1738 carcasses from 2020. The state veterinary services, bird clinics, wild bird rescue stations, zoos, wild parks, and academic institutes submitted whole bird carcasses or, in rare cases, various tissue material (mostly brain, liver, spleen, and heart) to the regional veterinary laboratories or to the national reference laboratory (NRL) for WNV at the Friedrich-Loeffler-Institut (FLI).

### 2.2. Molecular Investigations

To extract viral RNA from the frozen (−70 °C) coagulum/cruor and from organ samples, we used the RNeasy Mini Kit (Qiagen, Hilden, Germany), according to the manufacturer’s instructions. The analysis of the extracted RNA was performed with reverse transcription quantitative real-time PCR (RT-qPCR) assays specific for WNV as described by Eiden et al. [44] and for USUV as described by Jöst et al. [15]. For routine cases, an RT-qPCR with WNV-specific 5′NTR primers and probe [44] and a USUV RT-qPCR that targets the nonstructural protein 1 gene [15] were performed. To resolve doubtful results or to confirm the first detection of virus in new areas, a WNV RT-qPCR in the NS2Aregion [44] or a USUV RT-qPCR that target the NS5 gene [45] were used. Based on the NRL guidelines, quantification cycle (Ct) values <37 were regarded as positive, from 37 to 40 as possibly positive, and >40 as negative. In all RT-qPCR assays, positive controls with 10^3^ and 10^4^ WNV or USUV genome copies per reaction were included.

### 2.3. Sequencing and Phylogenetic Analysis

The extracted viral nucleic acid was reverse-transcribed and amplified using USUV-specific oligonucleotide primers (Supplemental Table S1) according to Eiden et al. [46], using SuperScript III One-Step RT-PCR System with Platinum Taq DNA Polymerase (Invitrogen, Thermo Fisher Scientific, Darmstadt, Germany) and the CFX96 Real-Time PCR Detection System (Bio-Rad Laboratories, Feldkirchen, Germany). The following thermal profile was used: reverse transcription at 45 °C for 15 min, Taq Polymerase activation at 95 °C for 3 min, 40 cycles of amplification (at 95 °C for 20 s, 55 °C for 30 s, and 68 °C for 1 min), and final elongation at 68 °C for 7 min. PCR products were subsequently purified via gel electrophoresis (1.5% agarose gel) and stained either using ethidium bromide or SYBR Safe DNA gel stain (Invitrogen). The gels were visualized with UV light or blue light, respectively. DNA bands of the expected size were excised from the gel and purified with the QIAquick Gel Extraction Kit (Qiagen) or the Wizard SV Gel and PCR Clean-Up System (Promega, Walldorf, Germany). Samples were sequenced via the TubeSeq service of Eurofins (Eurofins, Konstanz, Germany). The obtained partial sequences of the envelope protein (1261 nt) from the 61 analyzed samples were used to construct a phylogenetic tree. In a few cases, USUV sequences could not be obtained due to the low quality of the samples, particularly with higher Ct values (>30).

The Clustal W algorithm was used to align the sequences as implemented in MEGA v.11 software [47]. For estimation of pairwise genetic distances, substitution saturation analysis was performed in DAMBE v.7 [48], applying a GTR nucleotide substitution model. The best model of nucleotide substitutions (GTR+I+G4) was selected using jModeltest v.2 [49], and maximum likelihood trees were reconstructed using PAUP* v.4 [50]. The subtree-pruning-regrafting branch-swapping algorithm was applied to search for the heuristic tree. Reliability of the obtained tree topologies was performed by bootstrap testing (1000 replicates), and finalized trees were reconstructed with FigTree v.1.4.3 [51].

A separate tree was constructed with five partial genome sequences derived from the samples from Leipzig by the cooperation partner. These were sequenced using different USUV-specific primers [52] to obtain an envelope-coding gene sequence of 726 nucleotides, with an overlap region of only 474 nucleotides with the other primers.

### 2.4. Serological Investigations

Of the 2345 blood samples from wild birds, we could analyze 2281 samples by serological methods in two years; 952 sera were investigated in 2019 and 1329 sera in 2020. For primary serological screening, we performed a commercially available blocking ELISA (bELISA), which allowed the species-independent recognition antibodies against domain III of the envelope protein of WNV and USUV, following the manufacturer’s instructions (INgezim West Nile Compac, Ingenasa, Madrid, Spain). Samples were considered positive when the inhibition percentage (IP) was ≥40%, doubtful with IP of >30% to <40%, and negative with IP ≤ 40%. 

To confirm positive and doubtful ELISA results, reactive serum samples were also examined by differentiating virus neutralization tests (VNT), as described by Seidowski et al. [28] with minor modifications. The serum samples were tested against WNV lineage 2 (samples from 2019 with WNV strain Austria, GenBank accession HM015884, kindly provided by S. Revilla-Fernandez, AGES, Mödling, Austria, and samples from 2020 with WNV strain Germany, MH924836) as well as USUV strain Germany (HE599647) to clarify the ELISA results and to quantify cross-reacting antibodies among the Japanese encephalitis serogroup. Sera from experimentally infected animals or hyperimmune sera from vaccinated animals with known WNV and USUV antibody titers, as well as serum negative for flavivirus antibodies, were included as positive and negative controls, respectively. 

The neutralizing antibody titer (ND_50_) was determined as the reciprocal of the serum dilution that inhibited cytopathogenic effect in >50% of replicates and was calculated based on the Behrens–Kaerber method [53]. Serum samples with ND_50_ values equal to or above 10 were considered positive, and samples with ND_50_ values lower than 10 as negative. Wild birds were only scored positive for WNV-specific antibodies and used for the calculation of the seroprevalence rate if they had a reactive ELISA result in combination with a positive WNV VNT result and a negative (ND_50_ < 10) or significantly lower (fourfold lower) USUV titer. The same criteria for interpretation of VNT results were applied for the USUV serology. Use of WNV strain Austria or WNV strain Germany (both WNV lineage 2) had no influence on the specific neutralizing antibody titers (Supplemental Table S2).

Not all serum samples from the birds were investigated in the ELISA due to small volumes (<30 µL) in some submissions; in these cases, samples were analyzed directly in the VNTs. Additionally, a few serum samples were toxic for cells or hemolytic and thus were excluded from the evaluation. In addition, flavivirus species-specific antibody titers could not be determined for some sera, which were excluded from the calculation and considered as infections by an “unspecified flavivirus” (Supplemental Table S3). 

### 2.5. Statistical Analyses

Seroprevalence and 95% confidence intervals (95% CI) were calculated using R version 4.0.5 [54].

### 2.6. Maps

GIS analysis of the sampling sites and the results of WNV antibody positive birds, as well as the locations of the USUV-positive birds used for sequencing, was performed using ArcGIS ArcMap 10.8.1 (ESRI, Redlands, CA, USA) and open data from GeoBasis-DE/BKG 2020 [55].

### 2.7. Ethical Statement

Blood samples (first monitoring panel) came from birds from veterinary clinics or wild bird sanctuaries, falconry centers, wildlife parks, and zoos, where they had been collected for diagnostic purposes as part of a veterinary examination and in order to individually adapt treatment and prognosis. Some blood samples were taken immediately following euthanasia in birds with an infaust prognosis. Furthermore, blood sampling from sentinel ducks as part of an animal experiment was approved by the competent authority of the federal state of Mecklenburg-Western Pomerania, Germany (LALLF reference number 7221.3-2-006/19, approved 30 March 2019) on the basis of national and European legislation, namely, directive 2010/63/EU on the protection of animals used for scientific purposes. Tissue samples (second monitoring panel) were only taken from deceased birds that had been submitted for necropsy.

## 3. Results

With the partners of the nationwide wild bird surveillance network, 2345 blood samples were collected between 2019 and 2020. Samples in the panel represent 22 different bird orders (Table 1).

**Figure 1:**
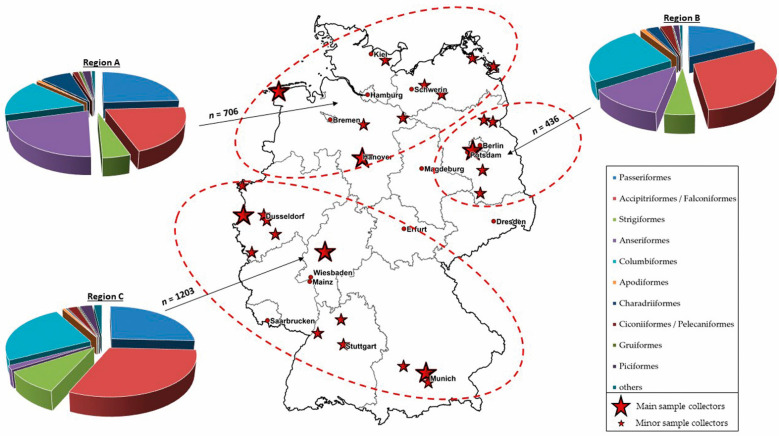
Total number (*n*) of blood samples collected in 2019 and 2020 and sampled bird orders per sampling region A to C. Region A: northern and central-western part of Germany; region B: eastern and central-eastern part of Germany; region C: central and southern parts of Germany (ellipses). The main sample collectors (big red stars) and the minor sample collectors (small red stars) of the wild bird network are shown for each region.

For a better interpretation of the molecular and serological results, taking in account to the geographical distribution, we divided our panel into three different regions of Germany. The northern and central-western part of Germany was referred to as region A, the eastern and central-eastern part as region B, and the central and southern parts of the country as region C. Figure 1 shows the total number of blood samples per sampling region and the sampled bird orders. The total number of blood samples from region A to C and the affiliation to the different bird orders and species can be found in the Supplemental Material (Tables S4–S6). 

### 3.1. Results of the RT-qPCR

Out of 2345 blood samples from wild birds (first monitoring panel/live bird monitoring), we could test 2312 samples (coagulum/cruor) in two years; 964 RNA specimens were analyzed by RT-qPCR in 2019 and 1348 in 2020. In 2019, USUV-RNA-positive birds were detected only in region B (*n* = 1) and C (*n* = 25), while in 2020, positive results for USUV were detected in blood samples (*n* = 16) in all three areas of Germany. Most birds that tested positive in these two years were Eurasian blackbirds (*n* = 11) and common wood pigeons (*Columba palumbus*, *n* = 11). 

In contrast, in both years, WNV-RNA-positive birds (*n* = 19) were only detected in region B. Northern goshawks (*Accipiter gentilis*, *n* = 15) and hooded crows (*Corvus corone cornix*, *n* = 3), as well as one grey heron (*Ardea cinerea*), were found to be affected. Interestingly, a goshawk from Berlin (region B) was co-infected with both flaviviruses, as confirmed by RT-qPCR.

All details of the 61 WNV or USUV RT-qPCR positive results from the live bird monitoring (first monitoring panel), including the affected bird species and their migration patterns, as well as the type of housing, are shown in Table 2. 

Our second monitoring panel comprised organ samples from 2976 deceased wild and captive birds; in detail, 1238 carcasses from 2019 and 1738 carcasses from 2020. In 2019, USUV-RNA-positive organs (mostly brain, liver, spleen, and heart) were detected in 151 dead birds, and 48 USUV-RNA-positive birds were detected in 2020. Details for bird species affected by USUV from the second panel (bird carcasses) can be found in Table 3.

Additionally, selected USUV-RNA-positive blood samples (first monitoring panel) and organs from bird carcasses (second monitoring panel) from 2019 and 2020 were included in the phylogeny studies; see Section 3.2.

### 3.2. Phylogenetic Analysis of USUV RNA Positive Birds

In order to determine the geographical distribution of the different USUV lineages in 2019 and 2020, blood and organ samples from all regions of the country were analyzed (in total, 5 blood samples and 61 tissue samples). Due to the variable occurrence of USUV in the regions during the two-year study period and the uneven availability of suitable sample material, this goal could not be achieved for all regions of Germany. In total, we received 43 partial sequences from the USUV outbreak in 2019, and 23 partial sequences from 2020. Detailed information on the phylogeny of the USUV partial sequences are presented in two phylogenetic trees in the Supplemental Figure S1A,B.

**Figure 2:**
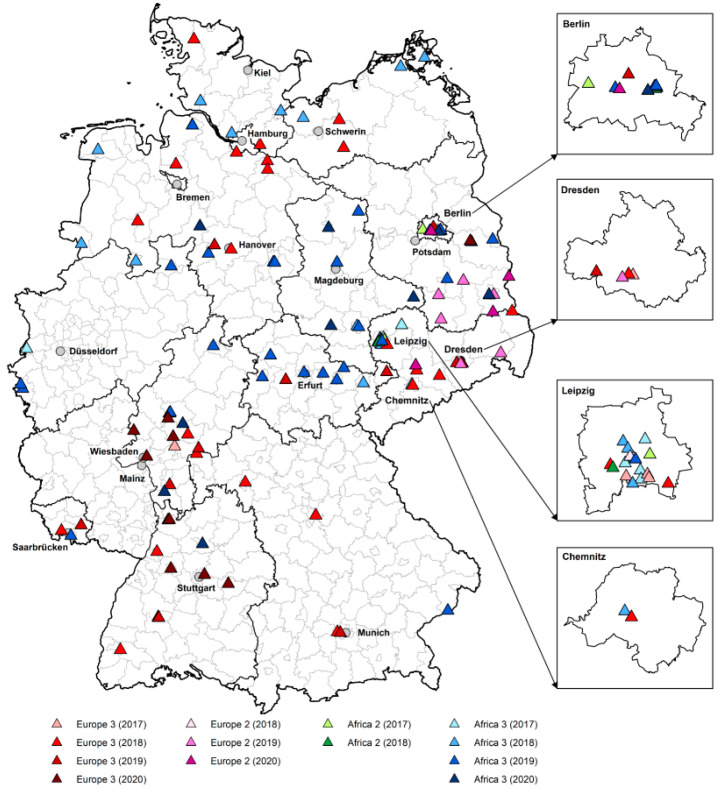
Circulation of the different USUV lineages in Germany from 2017 to 2020. The map includes 66 new partial USUV sequences from 2019 and 2020 (blood samples from birds in our first panel = live bird monitoring, and organ samples from the second panel = dead bird monitoring) and 60 sequences from a previous study from 2017 to 2018 published by Michel et al. [22], as well as six full-genome USUV sequences from birds of the co-infection study by Santos et al. [57]. The different USUV lineages are depicted as colored triangles: red = Europe 3, blue = Africa 3, green = Africa 2, and purple = Europe 2. Different shades of each of these colors indicate the years of detection of each USUV lineage.

Additional information about the bird species, localization, material of sequencing, and USUV lineage of the respective GenBank accession numbers is given in the Supplemental Table S7. In both years, USUV lineage Europe 3 was detected in wide parts of the country. USUV lineage Africa 3 was detected in all investigated regions as well. Interestingly, USUV lineage Europe 2 was found several times in the eastern part of the country, in different regions in Brandenburg and once in Saxony in 2019, and, apart from Saxony and Brandenburg, also once in Berlin in 2020. Surprisingly, USUV lineage Africa 2 was not detected in our study. The circulation of the different USUV lineages from this study in 2019 and 2020 were mapped in Figure 2 together with partial sequences from our previous bird study from 2017 and 2018 [22], as well as with six other USUV sequences from our co-infection study from 2018 and 2019 [57]. 

The WNV phylogenetic studies in dead birds, including also carcasses from 2019 (second monitoring panel in this study), have been published separately [38]. Furthermore, an extensive molecular survey based on the 2020 dead bird monitoring (second monitoring panel) was included in a separate study that examined the phylogeny of circulating WNV strains based on full genome sequences of 42 infected/dead birds and, additionally, 2 horses [58]. No further phylogenetic analyses were performed with the WNV-RNA-positive birds in the live bird blood monitoring (first panel) of 2019 to 2020.

### 3.3. Serological Results

**Figure 3:**
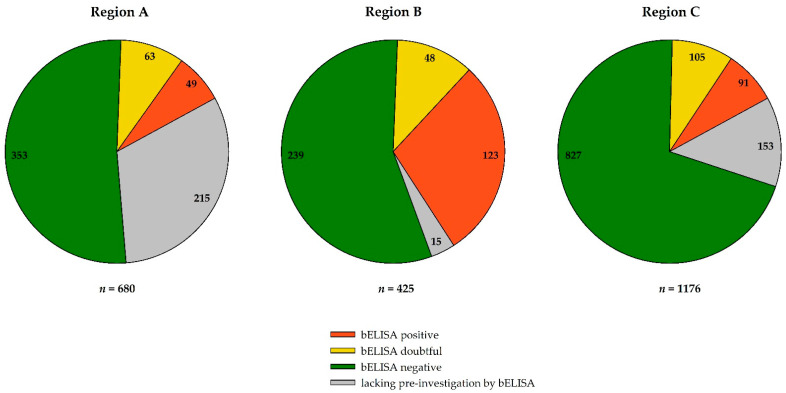
Distribution of blocking ELISA (bELISA) results across the three different sample regions (A–C) of Germany in 2019–2020. In the pie charts, the negative bELISA results are depicted in green, the reactive but doubtful results in yellow, and positive results in orange. The grey segments represent samples for which an initial bELISA screening was not possible due to small sample volume.

Of 2345 blood samples collected from wild birds, we received 952 sera in 2019 and 1329 sera in 2020. Of the serum samples investigated, 24.09% (112/465) in region A, 41.71% (171/410) in region B, and 19.15% (196/1023) in region C were reactive (doubtful and positive) in the bELISA (Figure 3), which is flavivirus-specific and is able to detect antibodies against both viruses.

Virus neutralization assays with reactive bELISA sera (*n* = 479), as well as with sera lacking prior bELISA testing (*n* = 383), revealed flavivirus species-specific reactivities and allowed an estimation of the overall seroprevalences for WNV and USUV in the three sample regions in 2019 and 2020. For USUV, the seroprevalence in region A was 4.13% (95% CI: 2.21–6.95) in 2019 followed by 4.11% (95% CI: 2.31–6.69) in 2020. In region B, the seroprevalence was 3.11% (95% CI: 1.01–7.10) in 2019 followed by a moderate increase to 7.20% (95% CI: 4.38–11.01) in 2020. In region C, the seroprevalence was 4.20% (95% CI: 2.58–6.41) in 2019 and 3.29% (95% CI: 2.09–4.88) in 2020. 

**Figure 4:**
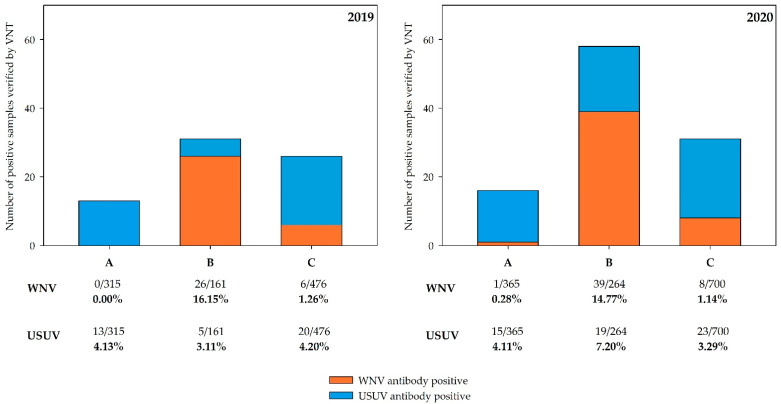
Number of positive bird serum samples verified by WNV and USUV VNT and the calculated seroprevalence for WNV and USUV in the three different study regions (A–C) in Germany in 2019 and 2020. The results for 2019 are shown on the left and for 2020 on the right. Serological results for 2019 include nine clinically affected free-ranging goshawks, which were published previously [56].

WNV seroprevalences differed from USUV seroprevalences. No specific WNV antibodies were detected in region A in 2019, and only one bird from this region (northern long-eared owl, *Asio otus*) showed a positive reaction by VNT in 2020 (ND_50_ 1/80; seroprevalence of 0.28% with 95% CI: 0.06–1.5). Region C had a low WNV seroprevalence in both years, specifically 1.26% (95% CI: 0.04–2.72) in 2019 and 1.14% (95% CI: 0.49–2.23) in 2020. However, seroprevalence in region B was consistently high in 2019 (16.15% with 95% CI: 10.82–22.76) and 2020 (14.77% with 95% CI: 10.72–19.64). The serological results are summarized in Figure 4 and detailed information on the corresponding VNT titers and all affected bird species are shown in the Supplemental Material (Tables S8 and S9). 

### 3.4. Regional Distribution of WNV- and USUV-Antibody-Positive Birds

In total, 95 USUV-antibody-positive birds were detected in all three regions (A–C) in Germany over the two-year investigation period. Except for region B in 2020 (7.20%), all other USUV seroprevalence rates were on a low level between 3.11% and 4.20%. The affected bird species were predominately Eurasian blackbirds (*n* = 14), common wood pigeons (*n* = 26), common buzzards (*Buteo buteo*, *n* = 10), and northern mallard ducks (*Anas platyrhynchos*, *n* = 4). The USUV serological results per regions and year, as well as the corresponding VNT titers, can be found in the Supplemental Material (Tables S8 and S9).

WNV-antibody-positive birds were primarily found in the eastern part of Germany (region B) (65 serologically positive results in both years combined). Here, primarily birds of prey (northern goshawks, *n* = 37, European kestrels (*Falco tinnunculus*), *n* = 2, common buzzards, *n* = 2, Eurasian sparrowhawks (*Accipiter nisus*), *n* = 2), passerine birds (hooded crows, *n* = 2, Eurasian blackbirds, *n* = 1, common magpie (*Pica pica*), *n* = 1), owls (northern long-eared owl, *n* = 2, Eurasian tawny owl (*Strix aluco), n* = 1), and pigeons (common wood pigeon, *n* = 10) tested positive by bELISA and VNT. Interestingly, in region C, in the center of Germany, few WNV-antibody-positive birds (resident and partial migrants) were detected in both years, namely, European kestrels (*n* = 6) and one saker falcon (*Falco cherrug*, kept for falconry), as well as two zoo birds (Kea (*Nestor notabilis*)) in Thuringia. In region A, only one owl (northern long-eared owl) showed a positive WNV reaction.

**Figure 5:**
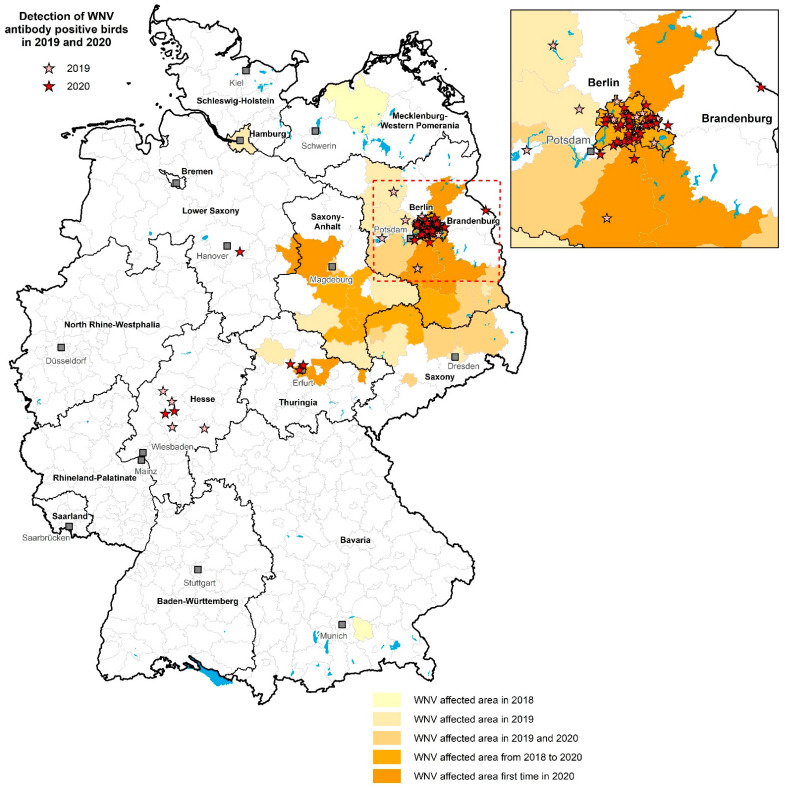
Detection of WNV-antibody-positive wild birds (resident and partial migrants) and their distribution in 2019 and 2020 in Germany. Light red stars represent the situation in 2019, dark red stars are the sites in 2020. The geographical distribution of the WNV-affected areas in Germany since the introduction of the virus in 2018 is shown in different shades of orange based on the extent of infection in each district, as defined by WNV-RNA-positive birds and WNV-RNA- and/or IgM-antibody-positive horses for the past three years (2018–2020). The figure includes nine serological results of ten free-ranging goshawks, clinically affected with WNV, which have already been published [56].

The detection of WNV antibodies in resident and partially migrant wild birds and their distribution in 2019 and 2020 in the country is depicted in Figure 5. Corresponding serological results for birds from the first panel are shown in Supplemental Tables S8 and S9. A large proportion of WNV-antibody-positive birds from region B died as a result of WNV infection due to typical clinical signs such as head tremor, ataxia, incoordination or apathy, or had to be euthanized due to a poor prognosis. Further details on clinical aspects of fatal WNV infection in free-ranging goshawks have already been described in a separate study [56]. Furthermore, one common wood pigeon showed high neutralizing antibody titers against both WNV (ND_50_ 1/240) and USUV (ND_50_ 1/320). 

## 4. Discussion

Continuous annual monitoring of the wild bird population is a useful tool to track the introduction of several new viruses. For USUV and WNV, we have used this tool in Germany for over ten years. This enabled us to detect the first entry of USUV in birds in the Upper Rhine valley in southwestern Germany in 2011 [16] and to monitor its annual spread and distribution. The spatial distribution over the first five years in the affected southwestern areas was consistent, only followed by a few sporadic cases in Berlin and Bonn [17,18,19]. In contrast to 2013–2015, there has been a strong numerical increase of USUV cases in 2016 as well as a regional spread to the federal states of North Rhine-Westphalia, Saxony, and Saxony-Anhalt [9,20]. The nationwide wild and zoo bird surveillance network for live birds (blood samples) also documented the massive spread of USUV over the whole country in 2018, and the first introduction of WNV in the same year [22,34]. Since then, there has been a stronger integration of investigations of dead wild and zoo birds from all regions of the country into the monitoring network.

**Figure 6:**
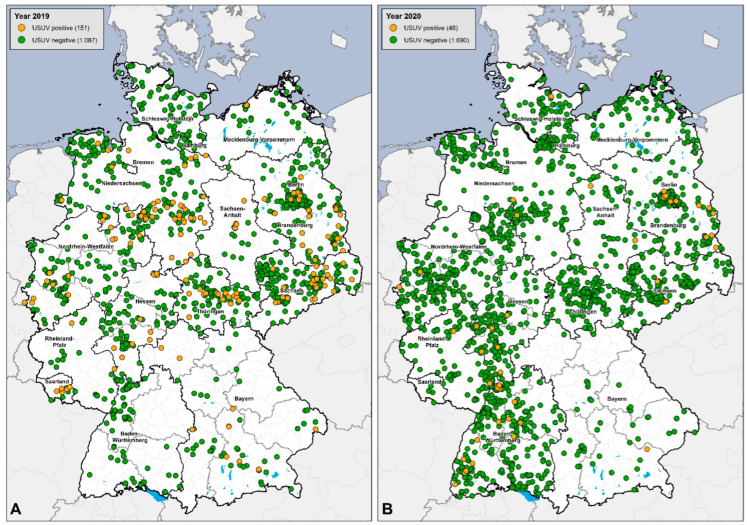
Database for USUV RNA detection in birds in Germany from 2019 and 2020. (**A**) Results from 2019: USUV-RNA-positive birds = 151 (orange dots) and USUV-RNA-negative birds = 1087 (green dots). (**B**) Results from 2020: USUV-RNA-positive birds = 48 (orange dots) and USUV-RNA-negative birds = 1690 (green dots).

To provide the public with a better overview of the nationwide WNV RNA and USUV RNA monitoring in dead birds (second panel in this study), a database was established at FLI (epidemiological group) to register and list all birds that tested positive or negative. The molecular results from each regional veterinary laboratory, as well as from the NRL, were included in this database. This makes it possible to track the expansion of USUV since 2019, even though this infection is not notifiable according to EU and German legislation. It shows that USUV was detected nationwide in 2019 (*n* = 151), but detections declined sharply in 2020 (*n* = 48) despite increased survey numbers (an increase of 500 birds compared to 2019) (Figure 6). The bird species affected by USUV (Table 3) corresponded to the already-known susceptible avian species in Germany [16,17,21,22]. 

With this in mind, a good proportion of birds affected by USUV (mostly blackbirds, *n* = 42) were selected for a phylogeny study with 66 partial sequences. Figure 2 shows the distribution of the different USUV lineages in birds during the two-year study period. As published in the previous monitoring study [22], the USUV lineages Europe 3 and Africa 3 spread throughout the whole country and were detected in numerous federal states. Interestingly, USUV lineage Europe 2 was detected multiple times in 2019 and 2020, but only in eastern areas of the country. Europe 2 was detected for the first time in 2018 in Leipzig (eastern Germany) [22]. The second detection occurred in 2019 in a wild bird (great tit (*Parus major*)) in Dresden (eastern Germany), which was also co-infected with WNV [57].

USUV Europe 2 circulates mainly in Austria, Hungary, and Italy [25], and our results suggest that this lineage is now endemic in Germany. An introduction into Germany via migration of infected birds from neighboring countries or via infected mosquitoes could have led to the first entry in 2018. Similar single introductions also occurred in Bonn for USUV Africa 3 [18] and in Berlin for USUV Africa 2 [19]. Surprisingly, the USUV Africa 2 lineage could not be detected in our sequencing panel in 2019 and 2020. Presumably, this lineage was not able to establish itself. This was also the case in our co-infection study [57], where two zoo birds from Berlin were initially detected with USUV lineage Africa 2 in 2018. However, in 2019, only two dead birds with USUV lineage Africa 3 infections were detected in the same zoo in Berlin. In addition, it was recently announced that in a retrospective study, USUV of lineage Africa 2 was also detected in a Eurasian blackbird in Hannover, in the northwestern part of Germany in 2018 [59]. However, no further data are available for this retrospective study for this region and lineage in 2019 and 2020. 

Similar results were found in the Netherlands, where a wide distribution of USUV lineages Europe 3 and Africa 3 with different sub-lineages has existed since 2016 [24]. Furthermore, a circulation of USUV between the Netherlands, Germany, and Belgium was assumed [24]. It is common knowledge that USUV has also been circulating in the eastern and southern neighboring countries, i.e., in Austria and the Czech Republic, for several years [11,60]. For Poland, the only evidence of USUV activity is based on the detection of specific antibodies in birds and horses [61,62]. Information on the circulating USUV lineages is available for the Czech Republic, with the circulation of multiple USUV lineages (Europe 1 and 2 and Africa 3) in Eurasian blackbirds and mosquitoes [63]. The same situation has been recently described for Austria [26,64]. Furthermore, for many years, USUV has been present in Italy, whereas lately, USUV lineages Europe 2 and Europe 4 have preferentially been circulating [25,65]. 

In the urban areas of Leipzig and Berlin, a circulation of four different USUV lineages has been found in 2017 and 2018 [22]. In addition, the circulation of WNV lineage 2 has been detected in these regions since 2018 [34,38]. On the one hand, the detection of WNV RNA and USUV RNA in mosquitoes [39,66] and the detection of infected mosquitoes in overwintering habitats in the eastern part has been described [66,67]. On the other hand, favorable climatic conditions with warm, humid springs and high temperatures in the summers led to a shorter extrinsic incubation period in infected mosquitoes and a faster transmission cycle between birds as hosts and mosquitoes as vectors, consecutively leading to a higher infection pressure [34,38]. Furthermore, the vector competence of indigenous *Culex pipiens* (biotypes *pipiens* and *molestus*) for the German WNV lineage 2 strain has been described recently [68], as well as for USUV [69]. This is reflected by the increased detection of USUV-infected birds and the high proportion of WNV-infected birds. Therefore, it is not surprising that in areas where both viruses are co-circulating, co-infections can occur. Recently, co-infections with WNV and USUV have been determined on the molecular level in five dead zoo birds collected in 2018 and 2019 in Berlin. Another co-infected wild bird has been found in Dresden in the eastern part of the country [57]. In our study, we also analyzed one blood sample from a diseased northern goshawk from Berlin (region B) in 2020, which was positive for WNV RNA and USUV RNA (Table 2). 

WNV as an important zoonosis has been present in Germany since the summer of 2018, and all circulating virus strains cluster in WNV lineage 2 [34,38]. Only a small epizootic occurred in the first year of detection, involving birds and horses, followed by a massive epizootic in the eastern part of Germany one year later [38]. Berlin and Leipzig turned out to be WNV infection hotspots. A similar scenario unfolded in 2020. Multiple goshawks were found with WNV infections. This is likely due to the high susceptibility of this species to a WNV infection, often associated with fatal neurological clinical signs [56]. Thanks to the well-functioning wild bird rescue system in Berlin and in the study regions, it was able to include these affected birds, including the goshawks, in the present study (Supplemental Tables S4–S6). In addition to positive WNV RNA findings in dead birds, there is clear evidence of WNV RNA in the blood samples from our live bird panel from region B (Berlin) (Table 2). Only a few sporadic cases occurred outside this region, with only one diseased Eurasian blackbird and one small passerine bird (*Prunella modularis*, a short distance migrant) in the northern part/region A (near Rostock in 2018 and in Hamburg in 2019), as well as two zoo birds in the south of the country/region C (near Munich in 2018) [34,38]. However, WNV did not prevail in these locations, as indicated by a lack of WNV in the indigenous mosquito population. The suboptimal climatic conditions at the time point of virus entry may be a possible reason for that outcome. This assumption is supported by the absence of WNV RNA in the avian blood samples from region A and C (Table 2).

Serological investigations revealed WNV-neutralizing antibodies in 65 out of 425 investigated serum samples from birds of region B (eastern part) in the time period 2019–2020, which is a considerably higher percentage than in a previous study [22]. Besides the detection of specific WNV antibodies in a few long-distance migratory birds, such as storks (*Ciconia* sp.), the majority of positive birds are resident or partial migrants with high titers of neutralizing antibodies (ND_50_ range from 1/10 to 1/5120, see Tables S8 and S9). Therefore, we can summarize that WNV has reached the resident bird population in region B and the virus spread in the local bird and mosquito population in an endemic cycle over the following years. It is remarkable that primarily birds of prey were detected as clearly serologically positive, most of all the goshawks. It is well known that several European species of birds of prey are susceptible to WNV infection [70,71,72,73,74,75,76]. Particularly, goshawks seem to be suitable as an indicator species to detect the beginning of a WNV infection wave in an area, as they are most susceptible to the virus [77,78]. Our serological results are linked to the finding that birds of prey can develop high titers of neutralizing antibodies after natural or experimental WNV infection [79]. The fact that a multiplicity of WNV-antibody-positive birds in region B belong to resident or partially migrant bird species indicates that WNV is circulating between mosquitoes and birds in the local bird population. These results are further supported by the fact that passerine birds (e.g., Eurasian blackbird, hooded crow, or magpie) and pigeons (e.g., common wood pigeon) were clearly detected as serologically positive for WNV antibodies by bELISA and VNT in the study. Furthermore, magpies or pigeons could be used as valuable sentinel species for the distribution of WNV in affected areas, as described for other countries [80,81]. All these results are in accordance with the known WNV endemic areas in the eastern part of the country and correspond to the evidence of WNV RNA in infected birds, horses, and humans [38,57]. Furthermore, a flavivirus double infection in one common wood pigeon was verified by VNT, as previously described for zoo birds in the area of Berlin [57]. The observed high neutralizing antibody titers against both WNV and USUV could be a rare event due to sequential or simultaneous infections as a consequence of a high infection pressure for both flaviviruses in region B. 

Interesting serological results from our study are depicted for regions A and C, with low levels of WNV seroprevalence (from 0.28% to 1.26%) combined with no detection of WNV RNA. However, the seroconverted birds (mainly birds of prey and one owl) showed a considerably high amount of neutralizing WNV antibodies (ND_50_ between 1/15 and 1/960, Figure 5, Supplemental Tables S8 and S9). This indicates an incipient spread of WNV to the west and south and calls for increased surveillance of birds as well as mosquitoes in these regions.

As represented by serology, USUV-specific antibodies were detected in approximately 3–7% of birds in the study, and USUV seroprevalence for Germany overall was similar (3.99% in 2019 with 95% CI: 2.84–5.44, and 4.29% in 2020 with 95% CI: 3.26–5.52). These USUV seroprevalences are actually slightly lower than in 2017 and 2018 [22] and are similar to 2015 [21]. Development of herd immunity with seroconversion rates above 50%, as observed in Austria after a massive spread of USUV in birds in 2001 [82], has not occurred in Germany yet.

A commercially available blocking ELISA (INgezim West Nile Compac, Ingenasa, Madrid, Spain) was used for primary serological screening, which is designed for the detection of WNV antibodies [83]. This ELISA is only partially specific for flaviviruses, and other non-WNV flavivirus antibodies are also detected [84]. However, with this ELISA it is possible to detect specific USUV antibodies, because USUV shares many structural and antigen characteristics with WNV. One study regarding blocking ELISA showed, on the basis of cross-reactions, that a partial detection of other flaviviruses, including USUV, is possible, but with unknown efficacy [84]. In contrast, we demonstrated that WNV- and USUV-antibody-positive bird sera from our selected panel were detected likewise by this ELISA within this study (Supplemental Table S10). Therefore, it is important to differentiate reactive ELISA results by VNT as the accepted gold standard for WNV- or USUV-specific antibodies, albeit not all ELISA results can always be distinguished this way [81,85]. In general, VNTs have a higher degree of specificity but are less sensitive than ELISAs [85], as both methods measure different serological parameters. Nevertheless, the staggered approach of ELISA testing first and then VNT confirmation of reactive results has proven to be a pragmatic approach for WNV and USUV serology. However, it is possible that some sera may be identified as USUV false negative by bELISA, and thus are not further investigated by VNT methods. This fact could be a limitation of this diagnostic approach.

Due to cross-reactivity, the circulation of known or as-yet-unknown flavivirus(es) in our regions cannot be excluded but is considered highly unlikely. Known flaviviruses that can induce cross-reactive antibodies, e.g., Louping ill virus [86] and Bagaza virus [87], as well as Meaban virus or Japanese encephalitis virus, have not been found in Germany yet (summarized by [88]). In addition, no unknown flavivirus was detected in the recent WNV seroprevalence survey in the German horse population from 2018–2020 [89]. 

## 5. Conclusions

Taken together, two USUV lineages (Europe 3 and Africa 3) were detected in 2019 and 2020 with a wide distribution throughout Germany, whereas USUV lineage Europe 2 only circulated in the eastern part of Germany. Despite its widespread occurrence, USUV dissemination was far from sufficient to induce herd immunity. 

For the first time, a high WNV seroprevalence (16.15% and 14.77%) in resident and partially migrant birds was observed in eastern Germany (region B), which correlates with the detection of WNV-RNA-positive live and deceased birds in this area. Interestingly, for the first time, signs of a further spread of WNV infections in western and southern directions were shown on a serological level, but currently without the detection of WNV RNA in birds in regions A and C.

Staggered testing by blocking ELISA followed by discriminatory VNTs is a useful approach for WNV and USUV serology in birds. However, improved assays for rapid serologic diagnosis of these flavivirus infections are certainly recommended. 

The German wild bird monitoring network is a useful tool to track the spread of WNV and USUV in the bird population. It is important to continue this program in the coming years and to extend its application to other relevant arboviruses or new emerging zoonotic viruses. 

## Figures and Tables

**Table 1:** Wild bird blood samples from Germany from 2019 to 2020 (first monitoring panel from live birds), grouped by taxonomic orders.

Order	2019	2020	Total
Passeriformes	232	327	559
Accipitriformes/Falconiformes	303	348	651
Strigiformes	86	81	167
Anseriformes	56	169	225
Columbiformes	169	346	515
Apodiformes	14	5	19
Charadriiformes	50	19	69
Ciconiiformes/Pelecaniformes	23	24	47
Gruiformes	5	3	8
Piciformes	20	34	54
Cuculiformes	1	0	1
Suliformes	1	1	2
Psittaciformes	1	4	5
Podicipediformes	1	0	1
Galliformes	7	3	10
Struthioniformes	0	1	1
Coraciiformes	2	0	2
Gaviiformes	0	1	1
Procellariiformes	1	0	1
Caprimulgiformes	1	0	1
unknown	4	2	6
Total	977	1368	2345

**Table 2:** Positive results of RT-qPCR among blood samples (first panel) from birds collected in three regions of Germany from 2019 to 2020 with the relevant flavivirus highlighted in red. The 2019 results include five WNV RNA positive samples from ten clinically affected free-ranging northern goshawks, which have already been reported [56].

Year	Region	Order	Common Name	Scientific Name	No. Tested Birds	Migration Pattern	Housing	WNV RT-qPCR No. Pos. (%)	USUV RT-qPCR No. Pos. (%)
2019	B	Accipitriformes	Northern Goshawk	*Accipiter gentilis*	18	R, P	wild	5 (27.8)	0 (0)
		Passeriformes	Eurasian Blackbird	*Turdus merula*	5	R, P	wild	0 (0)	1 (20)
			Hooded Crow	*Corvus cornix*	13	R, (S)	wild	1 (7.7)	0 (0)
		Pelecaniformes	Grey Heron	*Ardea cinerea*	5	R, P, S	wild	1 (20)	0 (0)
	C	Accipitriformes	Common Buzzard	*Buteo buteo*	34	R, P, S	wild	0 (0)	3 (8.8)
			Harris’s Hawk	*Parabuteo unicinctus*	14	zoo bird	captive	0 (0)	1 (7.1)
			Northern Goshawk	*Accipiter gentilis*	8	R, P	wild	0 (0)	1 (12.5)
			Steppe Eagle	*Aquila nipalensis*	4	zoo bird	captive	0 (0)	1 (25)
			European Honey Buzzard	*Pernis apicorus*	3	L	wild	0 (0)	1 (33.3)
		Columbiformes	Common Wood Pigeon	*Columba palumbus*	42	R, P, S	wild	0 (0)	6 (14.3)
		Passeriformes	Thrush	*Turdus* sp.	4	S, L	wild	0 (0)	1 (25)
			Eurasian Blackbird	*Turdus merula*	29	R, P	wild	0 (0)	4 (13.8)
			Carrion Crow	*Corvus corone*	23	R, (S)	wild	0 (0)	2 (8.7)
			House Sparrow	*Passer domesticus*	7	R	wild	0 (0)	1 (14.3)
		Pelecaniformes	Grey Heron	*Ardea cinerea*	6	R, P, S	wild	0 (0)	1 (16.7)
		Strigiformes	Eurasian Tawny Owl	*Strix aluco*	12	R	wild	0 (0)	2 (16.7)
			Great Grey Owl	*Strix nebulosa*	2	zoo bird	captive	0 (0)	1 (50)
In Total in 2019					964			7 (0.7)	26 (2.7)
2020	A	Passeriformes	Eurasian Blackbird	*Turdus merula*	29	R, P	wild	0 (0)	1 (3.4)
	B	Accipitriformes	Northern Goshawk	*Accipiter gentilis*	31	R, P	wild	10 (32.3)	1 (3.3)
		Columbiformes	Common Wood Pigeon	*Columba palumbus*	60	R, P, S	wild	0 (0)	3 (5)
		Passeriformes	Hooded Crow	*Corvus cornix*	32	R, (S)	wild	2 (6.3)	0 (0)
			Great Tit	*Parus major*	1	R, (P)	wild	0 (0)	1 (100)
	C	Accipitriformes	Harris’s Hawk	*Parabuteo unicinctus*	7	zoo bird	captive	0 (0)	1 (14.3)
			Northern Goshawk	*Accipiter gentilis*	10	R, P	wild	0 (0)	1 (10)
		Anseriformes	Mute Swan	*Cyngus olor*	2	R, P, S	wild	0 (0)	1 (50)
		Columbiformes	Common Wood Pigeon	*Columba palumbus*	165	R, P, S	wild	0 (0)	2 (1.2)
		Passeriformes	Eurasian Blackbird	*Turdus merula*	37	R, P	wild	0 (0)	5 (13.5)
In Total in 2020					1348			12 (0.9)	16 (1.2)

Note: R = resident species; P = partial migrants; S = short-distance migrants; L = long-distance migrants.

**Table 3:** Detailed information about bird species affected by USUV in 2019 and 2020 from the second panel (deceased birds/bird carcasses).

Year	Order	Common Name	Scientific Name	Migration Pattern	Housing	USUV RNA Positive/Tested
2019		Eurasian Blackbird	*Turdus merula*	R, P	wild	98/223
		Eurasian Blue Tit	*Parus caeruleus*	R	wild	1/6
		Thrush sp.	*Turdus* sp.	S, L	wild	3/18
		*True Finches* sp.	*Fringillidae*	n.d.	wild	2/14
	Passeriformes	European Greenfinch	*Carduelis chloris*	S	wild	1/35
		House Sparrow	*Passer domesticus*	R	wild	1/13
		Great Tit	*Parus major*	R, (P)	wild	2/6
		*Corvis* sp.	*Corvus* sp.	R, (P)	wild	1/6
		*Tits* sp.	*Parus* sp.	R, (P)	wild	2/6
		European Robin	*Erithacus rubecula*	R, P	wild	1/3
		Song thrush	*Turdus philomelos*	R, S	wild	5/12
		Common Starling	*Sturnus vulgaris*	R, P, S	wild	4/32
		Coal Tit	*Parus ater*	R, S	wild	2/3
		Great Grey Owl	*Strix nebulosa*	zoo	captive	9/15
		Snowy Owl	*Bubo scandiacus*	zoo	captive	3/17
	Strigiformes	Northern Hawk Owl	*Surnia ulula*	zoo	captive	1/4
		Eurasian Tawny Owl	*Strix aluco*	R	wild	1/6
		Northern Long-eared Owl	*Asio otus*	R, P, S	wild	1/2
		*Owls* sp.	n.d.	n.d.	captive	3/8
	Galliformes	Eurasian Capercaillie	*Tetrao urogallus*	zoo	captive	1/4
	Charadriiformes	Black-tailed Gull	*Larus crassirostris*	zoo	captive	2/8
	Anseriformes	Red-breasted Goose	*Branta ruficollis*	zoo	captive	1/2
	Columbiformes	*Pigeon* sp.	*Columba* sp.	n.d.	wild	1/70
	Unknown	Unknown species	n.d.	n.d.	captive/wild	5/101
In Total						151/(1238)
2020	Passeriformes	Eurasian Blackbird	*Turdus merula*	R, P	wild	24/158
	Accipitriformes	Blue Tit	*Parus caeruleus*	R	wild	5/156
	Passeriformes	Common Chaffinch	*Fringilla coelebs*	R, P	wild	2/33
		*Thrush* sp.	*Turdus* sp.	S, L	wild	4/22
	Accipitriformes	House Sparrow	*Passer domesticus*	R	wild	1/22
		Great Tit	*Parus major*	R, (P)	wild	2/72
	Anseriformes	*Tits* sp.	*Parus* sp.	n.d.	wild	1/20
		European Robin	*Erithacus rubecula*	R, P	wild	1/9
		Song Trush	*Turdus philomelos*	R, S	wild	1/8
		Great Grey Owl	*Strix nebulosa*	zoo	captive	2/6
		Mute Swan	*Cygnus olor*	R, P, S	wild	1/13
		*Penguin* sp.	*Spheniscus* sp.	zoo	captive	1/21
		Unknown species	n.d.	n.d.	captive/wild	3/177
In Total						48/(1738)

Note: R = resident species; P = partial migrants; S = short-distance migrants; L = long-distance migrants; n.d. not determined.

## Data Availability

The data that support the findings of this study are available in the main manuscript and in the Supplementary Material of this article. Genetic sequence data are publicly available in the GenBank repository.

## Supplementary Materials

The following are available online at https://www.mdpi.com/article/10.3390/microorganisms10040807/s1, Supplementary Materials Data shows: Figure S1A,B: Phylogenetic analysis of USUV strains detected in birds from Germany in 2019 and 2020, Table S1: USUV-specific primers used in this study, Table S2: Comparative analysis of the WNV antibody neutralization titers in positive reference sera by using two different WNV lineage 2 strains in VNT, Table S3: No differentiation between WNV and USUV by neutralization assays from wild bird blood samples was possible in regions A–C from the year 2019 and 2020, Table S4: Detailed register of the tested blood samples by bird orders and bird species from region A (northern and central-western part of Germany) from 2019 and 2020, Table S5: Detailed register of the tested blood samples by bird orders and species from region B (eastern and central-eastern part of Germany) from 2019 and 2020, Table S6: Detailed register of the tested blood samples by bird orders and species from region C (central and southern part of Germany) from 2019 and 2020, Table S7: Detailed information on the origin of phylogenetically analyzed USUV from wild and captive birds in the live and dead bird monitoring in 2019 and 2020, Table S8: WNV- and USUV-positive neutralization assay results from wild bird blood samples from regions A–C in 2019, Table S9: WNV- and USUV-positive neutralization assay results from wild bird blood samples from regions A–C in 2020, Table S10: Investigation of WNV and USUV antibody positive reference and bird sera by blocking ELISA (INgezim West Nile Compac) and VNT.
