# Supplementary material for: Spread of West Nile Virus and Usutu Virus in the German Bird Population, 2019–2020
Source: Microorganisms. 2022 Apr 12;10(4):807. doi: 10.3390/microorganisms10040807 (PMC9030481; doi:10.3390/microorganisms10040807)
Supplement: Supplementary file 1 [file microorganisms-10-00807-s001.zip › Figure S1A,B final 11-04-2022.pdf]

**Supplemental Figure S1:**

Phylogenetic analysis of USUV strains detected in birds from Germany in 2019 and 2020.

**A)** Partial envelope genome nucleotide sequences (1200 bp) sequenced by the Friedrich Loeffler Institute.

**B)** Partial nucleotide sequences for the envelope protein (800 bp) sequenced by the University of Leipzig.

Sequences are labeled by codes containing the GenBank accession number, year of sample collection and country of origin. The sequences from Germany described in this paper are highlighted in red.

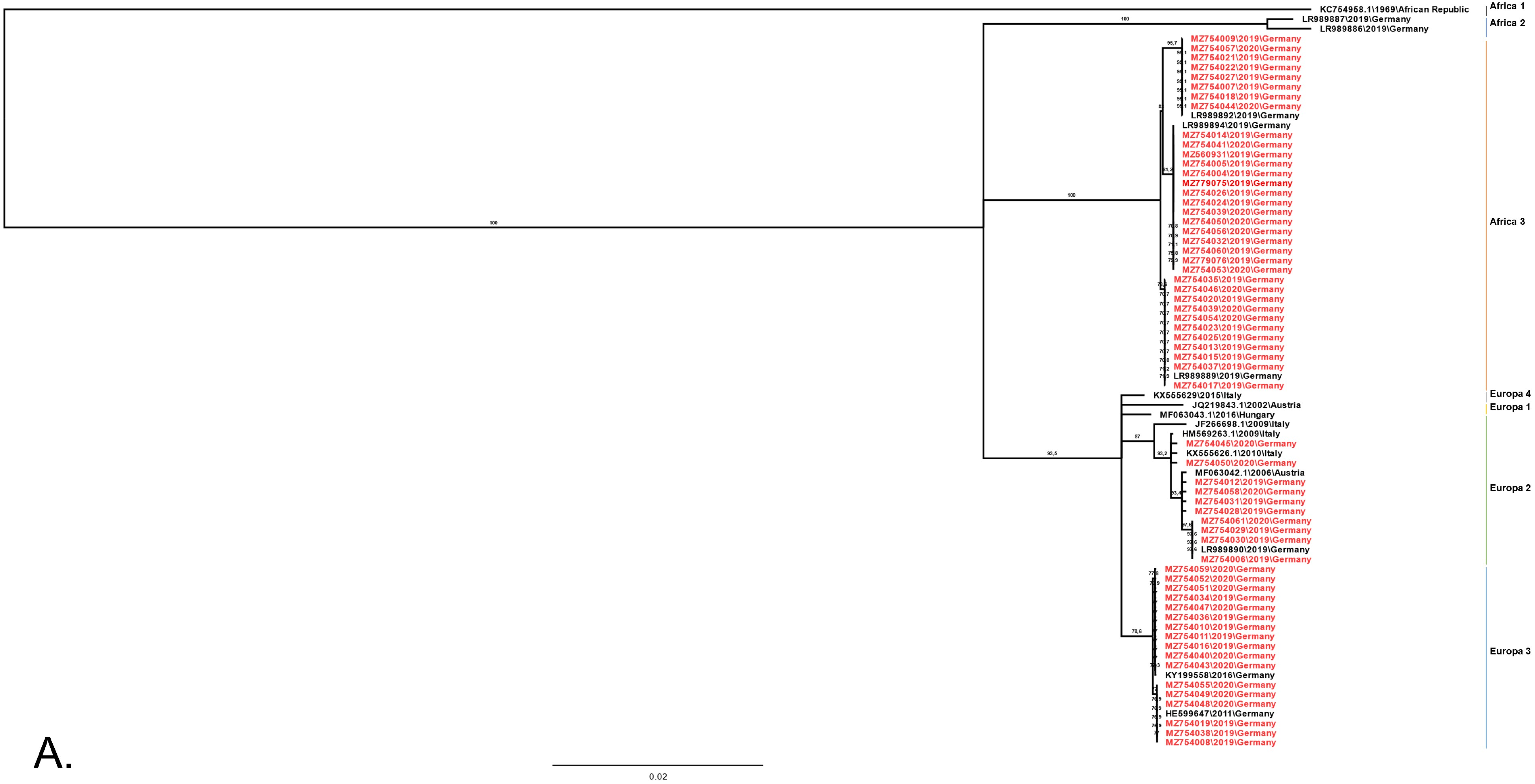

KC754958.1\1969\African Republic
LR989887\2019\Germany
LR989886\2019\Germany
MZ754009\2019\Germany
MZ754057\2020\Germany
MZ754021\2019\Germany
MZ754022\2019\Germany
MZ754027\2019\Germany
MZ754007\2019\Germany
MZ754018\2019\Germany
MZ754044\2020\Germany
LR989892\2019\Germany
LR989894\2019\Germany
MZ754014\2019\Germany
MZ754041\2020\Germany
MZ560931\2019\Germany
MZ754005\2019\Germany
MZ754004\2019\Germany
MZ779075\2019\Germany
MZ754026\2019\Germany
MZ754024\2019\Germany
MZ754039\2020\Germany
MZ754050\2020\Germany
MZ754056\2020\Germany
MZ754032\2019\Germany
MZ754060\2019\Germany
MZ779076\2019\Germany
MZ754053\2020\Germany
MZ754035\2019\Germany
MZ754046\2020\Germany
MZ754020\2019\Germany
MZ754039\2020\Germany
MZ754054\2020\Germany
MZ754023\2019\Germany
MZ754025\2019\Germany
MZ754013\2019\Germany
MZ754015\2019\Germany
MZ754037\2019\Germany
LR989889\2019\Germany
MZ754017\2019\Germany
KX555629\2015\Italy
JQ219843.1\2002\Austria
MF063043.1\2016\Hungary
JF266698.1\2009\Italy
HM569263.1\2009\Italy
MZ754045\2020\Germany
KX555626.1\2010\Italy
MZ754050\2020\Germany
MF063042.1\2006\Austria
MZ754012\2019\Germany
MZ754058\2020\Germany
MZ754031\2019\Germany
MZ754028\2019\Germany
MZ754061\2020\Germany
MZ754029\2019\Germany
MZ754030\2019\Germany
LR989890\2019\Germany
MZ754006\2019\Germany
MZ754059\2020\Germany
MZ754052\2020\Germany
MZ754051\2020\Germany
MZ754034\2019\Germany
MZ754047\2020\Germany
MZ754036\2019\Germany
MZ754010\2019\Germany
MZ754011\2019\Germany
MZ754016\2019\Germany
MZ754040\2020\Germany
MZ754043\2020\Germany
KY199558\2016\Germany
MZ754055\2020\Germany
MZ754049\2020\Germany
MZ754048\2020\Germany
HE599647\2011\Germany
MZ754019\2019\Germany
MZ754038\2019\Germany
MZ754008\2019\Germany
Africa 1
Africa 2
Africa 3
Europa 4
Europa 1
Europa 2
Europa 3
100
100
100
95,7
81,2
93,5
87
93,2
93,4
97,6
78,6
0.02
A.

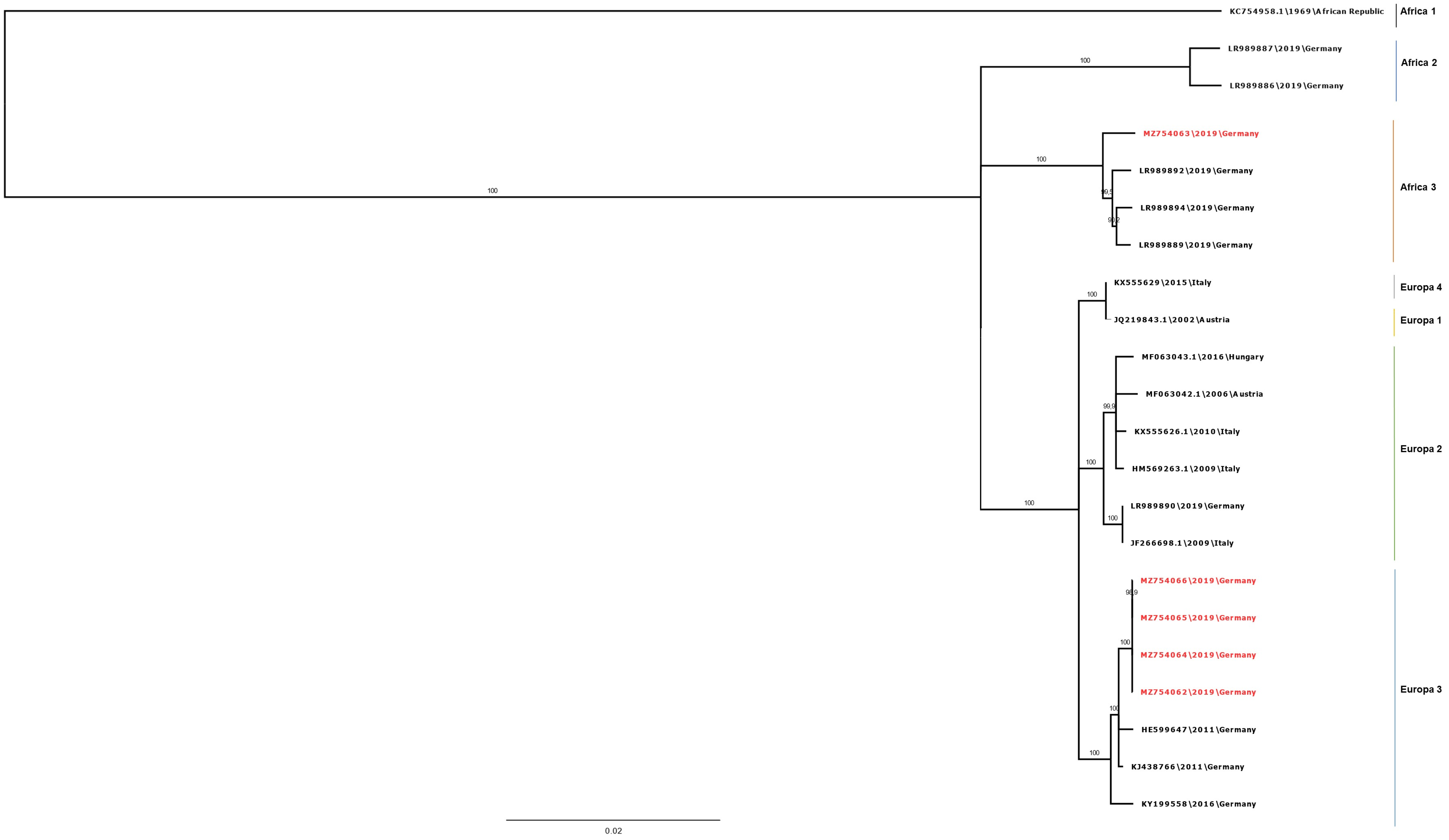

KC754958.1\1969\African Republic
Africa 1
LR989887\2019\Germany
LR989886\2019\Germany
Africa 2
MZ754063\2019\Germany
LR989892\2019\Germany
LR989894\2019\Germany
LR989889\2019\Germany
Africa 3
KX555629\2015\Italy
Europa 4
JQ219843.1\2002\Austria
Europa 1
MF063043.1\2016\Hungary
MF063042.1\2006\Austria
KX555626.1\2010\Italy
HM569263.1\2009\Italy
LR989890\2019\Germany
JF266698.1\2009\Italy
Europa 2
MZ754066\2019\Germany
MZ754065\2019\Germany
MZ754064\2019\Germany
MZ754062\2019\Germany
HE599647\2011\Germany
KJ438766\2011\Germany
KY199558\2016\Germany
Europa 3
100
99,5
99,9
0.02


B.
